# Gait asymmetry across frailty-status groups in older adults: clinical and biomechanical evidence from 3D gait analysis

**DOI:** 10.1080/07853890.2026.2702656

**Published:** 2026-07-14

**Authors:** Jiaxin Gao, Xiaohua Ke, Qingping Su, Peiyuan Zhuo, Yaping Li, Sheng Huang, Xinya Lan, Cai Jiang, Zhonghua Lin

**Affiliations:** a Rehabilitation Medicine Center, Fuzhou University Affiliated Provincial Hospital, Fuzhou, China; b Department of Rehabilitation Medicine, Shanghai Fourth People’s Hospital, School of Medicine, Tongji University, Shanghai, China; c Shengli Clinical Medical College of Fujian Medical University, Fuzhou, China; d Department of Complementary Medicine, University of Johannesburg, Johannesburg, South Africa

**Keywords:** Frailty, gait asymmetry, three-dimensional gait analysis, older adults

## Abstract

**Background:**

Gait asymmetry is a sensitive marker of age-related neuromuscular dysfunction, but its multidimensional profiles across frailty stages remain unclear. This study characterized spatiotemporal, kinematic, and kinetic gait asymmetry across frailty-status groups.

**Methods:**

A total of 150 older adults (Non-frail = 49, Prefrail = 80, Frail = 21) underwent 3D gait analysis. Fifty-one gait asymmetry features spanning spatiotemporal parameters, joint kinematics, and kinetics were derived. Group differences were examined using Kruskal–Wallis tests and pairwise Mann–Whitney U tests with Benjamini–Hochberg false discovery rate (FDR) correction.

**Results:**

Nine of 51 features showed at least one significant pairwise difference after within-feature FDR adjustment. Compared with non-frail participants, prefrail participants exhibited greater asymmetry in single-support time (*p* = 0.033) and stride length (*p* = 0.001). Frail participants showed greater asymmetry than non-frail participants in stride length (*p* = 0.020) , hip flexion (*p* = 0.025), knee flexion (*p* = 0.019), hip external rotation (*p* = 0.026), knee flexion moment (*p* = 0.027), and knee eccentric power (*p* = 0.040). Compared with prefrail participants, frail participants also demonstrated greater asymmetry in hip flexion, hip external rotation, knee flexion moment, and knee eccentric power.

**Conclusions:**

Gait asymmetry differed across frailty-status groups. Prefrail participants mainly showed feature-level differences in spatiotemporal asymmetry, whereas frail participants showed additional feature-level differences involving hip/knee kinematic and knee kinetic asymmetry. These findings describe exploratory, domain-informed gait asymmetry patterns associated with frailty status. These cross-sectional associations require longitudinal validation before prognostic or clinical monitoring utility can be inferred.

Trial registration: Chinese Clinical Trial Registry, ChiCTR2300073905.

## KEY MESSAGES

1. Gait asymmetry showed cross-sectional differences across frailty-status groups, with feature-level differences distributed across spatiotemporal, kinematic, and kinetic domains.
2. In prefrail older adults, observed differences were mainly concentrated in spatiotemporal asymmetry, including stride length and single-support time.
3. In frail older adults, observed differences involved additional hip/knee kinematic and knee kinetic asymmetry outcomes; these findings should be interpreted as exploratory associations requiring longitudinal and external validation.

## 1. Introduction

With the rapid acceleration of population aging, frailty, which is characterized by reduced physiological reserve and multisystem impairment, has become one of the central issues in geriatrics and rehabilitation medicine [1–3]. Frailty is strongly associated with an increased risk of falls, hospitalization, disability, and mortality, and is frequently accompanied by progressive deterioration in physical function and performance of activities of daily living [3–7]. Therefore, identifying individuals at elevated risk before substantial functional deterioration occurs, objectively characterizing frailty-related functional changes, and providing a basis for subsequent intervention have become major priorities in both clinical practice and research [2,7].

Gait is a comprehensive indicator of lower-limb function, neuromuscular control, and overall motor capacity, and therefore holds unique value in the assessment of frailty [8–11]. Previous studies have shown that reduced gait speed, shorter step length, and increased gait variability are closely associated with frailty, falls, and decline in independence in activities of daily living [12–16]. As a result, ‘gait assessment’ has increasingly been incorporated into frailty screening and comprehensive geriatric assessment in recent years [11,17]. However, conventional gait assessment has predominantly focused on ‘global’ metrics such as speed and step length [10,11,13,15,17], with relatively limited attention to interlimb coordination and gait asymmetry.

Under normal conditions, the two lower limbs should maintain relative coordination and balance in terms of support, swing, and load distribution [18]. When neuromuscular function declines or compensatory strategies increase, this balance is often disrupted, leading to asymmetry across multiple levels, including support time, step length, joint angles, and joint moments [18,19]. Compared with global gait measures such as gait speed, gait asymmetry quantifies relative interlimb differences in motor control and mechanical load distribution and may capture information not fully reflected by conventional gait parameters [20,21]. Clinical observations also suggest that some older adults exhibit gait instability, weight-shifting tendencies, or unequal bilateral loading before marked gait slowing becomes apparent, indicating that gait asymmetry may be closely related to frailty-associated gait impairment [22,23]. Therefore, gait asymmetry warrants particular attention as a potentially informative measure for characterizing frailty-associated gait impairment and for identifying candidate features for further longitudinal evaluation.

However, evidence regarding the relationship between gait asymmetry and frailty remains insufficient. Previous studies have primarily focused on global gait parameters, such as gait speed and step length, or on a limited number of spatiotemporal asymmetry measures [24,25]. Studies using three-dimensional gait analysis to concurrently evaluate asymmetry across spatiotemporal, multi-joint kinematic, and kinetic domains remain relatively scarce. In particular, few studies have systematically compared multidimensional gait asymmetry patterns among non-frail, prefrail, and frail older adults within the same analytical framework. Consequently, it remains unclear whether differences in gait asymmetry across frailty-status groups are primarily confined to global gait rhythm and support patterns or extend to joint-level movement coordination and mechanical load distribution.

Three-dimensional gait analysis enables the simultaneous acquisition of multi-joint, multi-planar kinematic characteristics and kinetic measures, including joint moments, joint power, and ground reaction forces, thereby providing a more comprehensive basis for characterizing the biomechanical features of gait impairment across frailty-status groups [26]. Accordingly, the present study classified older adults as non-frail, prefrail, or frail using the Fried frailty phenotype [27] and applied three-dimensional gait analysis to derive 51 gait asymmetry indices spanning spatiotemporal, multi-joint kinematic, and kinetic domains. The objectives of this study were to (1): quantify and compare multidimensional gait asymmetry across non-frail, prefrail, and frail older adults (2); determine whether cross-sectional differences in gait asymmetry across frailty-status groups extend from spatiotemporal measures to multi-joint kinematic and kinetic features; and (3) describe which gait asymmetry indices showed exploratory cross-sectional associations with frailty status and may warrant further evaluation in future longitudinal studies.

## 2. Method

### 2.1. Study design and participants

This was a single-center observational cross-sectional study based on baseline data from participants enrolled in a registered randomized controlled trial (Chinese Clinical Trial Registry: ChiCTR2300073905). The present analysis included baseline assessments only and did not evaluate intervention effects or longitudinal changes. The study was conducted from August 2023 to July 2025 at the Provincial Hospital Affiliated to Fuzhou University in Fuzhou, Fujian Province, China, which was the sole study site. The study was reported in accordance with the Strengthening the Reporting of Observational Studies in Epidemiology (STROBE) statement [28]. The study protocol was approved by the Ethics Committee of the Provincial Hospital Affiliated to Fuzhou University (Ref: K2022-09-029), and written informed consent was obtained from all participants before enrollment.

Older adults across the frailty spectrum were recruited from the Departments of Rehabilitation Medicine and Geriatric Medicine at the Provincial Hospital Affiliated to Fuzhou University. Participants included both outpatients and community-dwelling volunteers. Before enrollment, all potential participants were screened by trained professionals according to the prespecified eligibility criteria.

The inclusion criteria were as follows:

1. Age 60–80 years.
2. completed frailty assessment and could be stratified according to prespecified criteria.
3. Ability to walk independently for at least 10 m without physical assistance from another person.
4. Ability to understand and follow standardized instructions and to complete the gait assessments safely.

The exclusion criteria were as follows:

1. Neurological disorders or residual neurological deficits that could clearly and independently affect gait or gait symmetry, such as post-stroke hemiparesis, Parkinson’s disease, cerebellar ataxia, clinically significant peripheral neuropathy, or other neurologically based gait abnormalities.
2. Major musculoskeletal or orthopedic conditions that could substantially affect lower-limb loading, alignment, or coordination, such as a history of hip or knee arthroplasty, major lower-limb surgery with persistent functional impairment, obvious lower-limb deformity, or previous lower-limb fracture with ongoing significant effects on gait.
3. Obvious pain during walking that had already altered the natural gait pattern.
4. Acute cardiopulmonary disease, unstable medical conditions, or other conditions that made gait testing unsafe or inappropriate.
5. Severe visual or hearing impairment that would preclude safe completion of the assessments.
6. Acute illness or acute exacerbation of a chronic disease at the time of assessment.

A total of 150 participants completed all assessments and were included in the final analysis.

### 2.2. Frailty assessment and grouping

Frailty status was assessed using the Fried frailty phenotype [27,29,30], which consists of the following five components:

1. Unintentional weight loss: Participants were asked whether they had experienced unintentional weight loss (i.e. not due to dieting or exercise) during the past 12 months. This criterion was considered present if the participant reported unintentional weight loss ≥4.5 kg or ≥5% of body weight in the prior year;
2. Exhaustion: Exhaustion was assessed using the two items from the Center for Epidemiologic Studies Depression Scale (CES-D) [31] used in the original phenotype (‘I felt that everything I did was an effort’ and ‘I could not get going’). Each item was rated by frequency during the past week (0–3). The exhaustion criterion was considered present if the participant responded 2 (a moderate amount of the time, 3–4 days) or 3 (most of the time) to either item;
3. Low physical activity: Weekly energy expenditure (kcal/week) was calculated according to the China Leisure Time Physical Activity Questionnaire (CLTPAQ) [32] algorithm: MET × times/week × minutes/time × body weight (kg)/60. Weekly kcal expenditure was ranked within each sex, and participants in the lowest 20% (sex-specific bottom quintile) were scored as meeting the low physical activity criterion [33];
4. Slowness: Walking 15 feet (4.57m) at usual pace. Slowness was considered present if the time exceeded the sex- and height-stratified cutoffs: men ≤173 cm/women ≤159 cm: ≥7s; men >173 cm/women >159 cm: ≥6s;
5. Weakness: Weakness was assessed by dominant-hand grip strength using a handheld dynamometer. Participants performed three maximal trials; the maximum value was used for phenotype classification. Weakness was considered present if grip strength was below the sex- and BMI-stratified cutoffs: Men: BMI ≤24: ≤29 kg; 24.1–26: ≤30 kg; 26.1–28: ≤30 kg; >28: ≤32 kg. Women: BMI ≤23: ≤17 kg; 23.1–26: ≤17.3 kg; 26.1–29: ≤18 kg; >29: ≤21 kg.

Participants were categorized into three groups according to the number of criteria met: those meeting 0 criteria were classified as non-frail; those meeting 1–2 criteria were classified as prefrail; and those meeting ≥3 criteria were classified as frail. In the final sample, 49 participants were non-frail, 80 were prefrail, and 21 were frail.

### 2.3. Data collection and assessment of functional status

All data were collected by trained research staff following a standardized protocol. General demographic and clinical information included age, sex, height, weight, body mass index (BMI), educational level, number of chronic conditions, and number of long-term medications. Functional status was assessed using the following scales and objective tests:

1. Handgrip strength [34]: Maximal isometric grip strength was measured using a handheld dynamometer. Participants were seated with the shoulder adducted and in a neutral rotation position, the elbow flexed at approximately 90°. Under standardized instructions and verbal encouragement, participants performed three maximal voluntary grip trials with the dominant hand, with an inter-trial rest interval of ≥30–60 s to minimize fatigue effects. The maximum value of the three trials was used as the final grip strength.
2. Timed Up and Go Test (TUGT) [35]: The TUGT was used to assess dynamic balance, gait speed, and fall risk. Participants were instructed to perform the test at a self-selected comfortable and safe pace: standing up from a chair, walking to a floor marker 3 m ahead, turning around (turning direction was not standardized and participants turned according to their natural preference), walking back to the chair, and sitting down. No physical assistance was provided during the test. One practice trial (not timed) was performed before a single timed trial, and the total completion time was recorded [36]. As a clinical reference, a time <10 s indicates essentially normal mobility, 10–19 s indicates independent mobility, 20–29 s suggests that assistance may be needed for some daily activities, and ≥30 s indicates severely limited mobility [35,37].
3. Short Physical Performance Battery (SPPB) [38]: The SPPB is a standardized tool for evaluating lower extremity physical performance. It consists of three components: standing balance tests, usual gait speed over a short distance, and repeated chair stand tests. The total score ranges from 0 to 12, with higher scores indicating better lower limb function.
4. Mini Nutritional Assessment-Short Form (MNA-SF) [39]: The MNA-SF was used to screen for malnutrition and risk of malnutrition in older adults. It contains six items covering food intake over the past three months, recent weight loss, mobility, acute disease or psychological stress, neuropsychological problems, and BMI (or calf circumference). A total score ≤7 indicates malnutrition, 8–11 indicates risk of malnutrition, and 12–14 indicates normal nutritional status.
5. Geriatric Depression Scale-Short Form (GDS-15) [40]: The GDS-15 is a self-report questionnaire for assessing depressive symptoms in older adults, consisting of 15 yes/no items. A total score of 0–4 is considered non-depressed, 5–9 suggests possible depressive symptoms, and 10–15 indicates more severe depressive symptoms.
6. Athens Insomnia Scale (AIS) [41]: The AIS is an 8-item self-report scale used to assess sleep quality and insomnia severity over the past month. Items cover sleep induction, night-time awakenings, early morning awakening, total sleep duration, overall sleep quality, and daytime functioning. A total score <4 indicates normal sleep, 4–6 suggests possible insomnia, and >6 indicates the presence of insomnia of varying severity.

### 2.4. Three-dimensional motion capture system

Gait data were collected using a SMART-DX 400 infrared three-dimensional motion capture system (BTS Bioengineering, Milan, Italy). The system comprised eight infrared cameras (200 Hz), two time-synchronized video cameras (BTS eVixta, 50 Hz), four force plates (BTS P6000D, 1000 Hz), and a dedicated acquisition and processing workstation. Kinematic and ground-reaction-force data were hardware-synchronized during acquisition and processed jointly in BTS SmartClinic.

### 2.5. Experimental setting and gait task

The experiment was conducted in the gait analysis laboratory of the Department of Rehabilitation Medicine at the Fuzhou University Affiliated Provincial Hospital. A 6 m straight walkway was used, with approximately 1 m reserved at each end to allow a natural transition after gait initiation and natural preparation for deceleration before the end of the walkway, thereby ensuring the capture of steady-state walking data. Participants were not instructed to intentionally accelerate or decelerate; instead, they walked barefoot at a self-selected, comfortable, and safe speed on a level surface. Only gait cycles corresponding to the middle steady-state walking segment were selected for three-dimensional gait analysis. Before formal data collection, participants performed 2–3 familiarization trials, followed by five formal walking trials.

### 2.6. Motion capture and force data acquisition procedures

 

1. Environment preparation: Curtains and strong light sources were closed or switched off, and reflective objects were removed from the capture area to ensure marker detection quality and participant comfort;
2. System calibration: The connections between the infrared cameras, synchronized video cameras, force plates, and the workstation were checked. Spatial calibration of the camera system and zeroing/accuracy calibration of the force plates were performed according to the manufacturer’s instructions to ensure accurate kinematic and kinetic measurements;
3. Participant preparation and anthropometrics: Participants walked barefoot and wore standardized tight-fitting clothing (a fitted vest and shorts) provided by the research team to protect privacy while minimizing marker occlusion. Height, body mass, lower-limb length, pelvic height, pelvic width, knee width, and ankle width were measured and recorded for individualized model construction and offset corrections;
4. Marker placement and quality control: Reflective markers were placed according to the Davis–Heel [42] lower-limbmodel on predefined anatomical landmarks. A total of 22 markers were used, including 18 skin-mounted markers and 4 strap-mounted cluster markers. Specifically, skin-mounted markers were placed on: the seventh cervical spinous process (C7) - 1, bilateral acromion processes - 2, bilateral anterior superior iliac spines (ASIS) - 2, the midpoint of the line connecting the bilateral posterior superior iliac spines (PSIS) - 1, bilateral greater trochanters - 2, bilateral lateral femoral epicondyles - 2, bilateral fibular heads - 2, bilateral lateral malleoli - 2, bilateral fifth metatarsophalangeal joints - 2, and bilateral heels - 2. Four strap-mounted markers were positioned at the midpoints of the greater trochanter–lateral femoral epicondyle segments (bilateral thighs) and the midpoints of the fibular head–lateral malleolus segments (bilateral shanks) using elastic straps. To reduce clothing-related artifacts, small openings were made at key pelvic landmarks (e.g. ASIS and greater trochanter) to allow direct skin attachment; markers were reinforced with medical-grade adhesive tape to minimize wobble and detachment. Marker placement was performed by two assessors who had completed standardized training and passed internal competency assessment. For consistency, each participant’s markers were placed by a single assessor. The two assessors conducted regular calibration sessions every two weeks with periodic cross-checking of landmark identification and placement procedures;
5. Static calibration trial: Participants stood in a natural upright posture with feet shoulder-width apart and arms relaxed at their sides for several seconds. A static trial was recorded to establish the three-dimensional skeletal model and joint coordinate systems. After marker placement, the static trial and a brief walk-through check were used to confirm anatomically appropriate marker positions, secure attachment, and absence of occlusion; if any marker was found to be loose, occluded, or displaced, it was immediately re-attached and the verification steps were repeated;
6. Dynamic gait trials: After static calibration, participants walked back and forth along the 6-m walkway five times at a self-selected comfortable speed. Before formal data collection. Research staff continuously monitored participant safety and data quality. Trials were discarded if participants reported obvious discomfort, exhibited atypical gait patterns, or failed to place the foot correctly on the force plates. At least three valid trials were required; if fewer than three valid trials were obtained, additional trials were collected to meet the minimum data-quality requirement. When more than three valid trials passed quality control, three trials were selected for subsequent analysis according to predefined criteria (clear marker trajectories, good signal quality, and a gait pattern closest to the participant’s usual walking).

### 2.7. Biomechanical modeling

A biomechanical model was constructed in BTS SMART-Clinic using the ‘DAVIS Heel: Multi-factor Gait Analysis’ protocol, which is based on the conventional Davis gait modeling framework [42,43]. Within this framework, the lower limb is modeled as a rigid-body chain comprising the pelvis, thigh, shank, and foot segments, and joint motion is described as the relative rotation between adjacent segments.

Segment coordinate systems were established during the static calibration trial using anatomical landmarks together with strap-mounted cluster markers, and were tracked during dynamic walking using the corresponding technical markers. The pelvic coordinate system was defined using the bilateral ASIS markers and the midpoint of the line connecting the PSIS markers, and a right-handed orthonormal axis system was constructed *via* vector definition and orthogonalization. For the thigh and shank, anatomical reference frames were defined during static calibration based on the corresponding anatomical markers (greater trochanter, lateral femoral epicondyle, fibular head, and lateral malleolus). The fixed transformation of each strap-mounted cluster relative to its anatomical reference frame was then computed. During dynamic walking, segment orientation was tracked frame-by-frame primarily using the thigh and shank cluster markers, and mapped to the anatomical reference frames using the fixed transformations obtained from static calibration, thereby reducing the influence of soft-tissue artefact on segment orientation estimation. Foot orientation was represented using the Davis model foot vector. Specifically, a heel–toe vector defined by the heel marker and the fifth metatarsophalangeal joint marker was used to describe the foot heading in the horizontal plane and to compute foot rotation and the foot progression angle.

Joint center estimation followed the Davis model [42]. The hip joint center was determined within the pelvic coordinate system using a regression-based method, with pelvic width, pelvic height, lower-limb length, and body height as input parameters. The knee and ankle joint centers were defined during static calibration based on the lateral bony landmarks and were then medially offset along the segment mediolateral direction using the measured knee width and ankle width, with additional correction applied for positional bias introduced by marker size.

During dynamic walking trials, joint centers were updated frame by frame by mapping the joint-center position vectors obtained from static calibration to the global coordinate system *via* rigid-body transformations based on segment pose, and these updated joint centers were used for joint angle computation and inverse dynamics. Joint angles were computed from the relative orientation between adjacent segment coordinate systems and decomposed using a y–x–z Cardan/Euler rotation sequence to obtain flexion–extension, adduction–abduction, and internal–external rotation. Static calibration trial data were used as baseline offsets for angle processing. Inverse dynamics were implemented using a linked-segment Newton–Euler approach. Using ground reaction forces (GRF) and center of pressure synchronized with kinematics, together with segment inertial parameters estimated from anthropometric data, net joint moments and powers at the hip, knee, and ankle were calculated.

### 2.8. Signal processing

Gait cycle processing was performed using BTS proprietary software (Smart Clinic). Marker trajectories and force-plate signals were filtered using a dual-pass fourth-order low-pass Butterworth filter, with cut-off frequencies set to 6 Hz and 50 Hz, respectively [44,45]. Gait events were identified from the filtered vertical GRF: heel strike (HS) was defined when vertical GRF rose above 10 N, and toe-off (TO) was defined when vertical GRF fell below 10 N [46]. Each gait cycle was defined as the interval between two consecutive ipsilateral HS events and was time-normalized to 0–100% of the gait cycle for waveform reporting and statistical analyses. Peak metrics were derived from the per-cycle, time-normalized waveform data exported from SmartClinic. For each valid gait cycle, the maximum (MAX) and minimum (MIN) values over the full cycle were extracted as peak representations (for example, peak knee flexion was defined as the maximum knee flexion–extension angle, whereas peak knee extension was defined as the minimum value of the same waveform; the same approach was applied to joint moments, joint power, and each GRF component). Kinetic variables were normalized to body mass or body weight: joint moments and joint power are reported in N·m/kg and W/kg, respectively, and GRF variables are reported as a percentage of body weight (% body weight).

### 2.9. Calculation of gait asymmetry indices

This study focused on gait asymmetry and derived a total of 51 asymmetry indices, covering three domains (1): Spatiotemporal parameters (2); Kinematic parameters: Asymmetry indices of peak joint angles in the sagittal, frontal, and transverse planes for major lower-limb joints (hip, knee, ankle), including flexion/extension, abduction/adduction, and rotation (3); Kinetic parameters: Asymmetry indices of peak hip, knee, and ankle joint extensor and flexor moments, joint power, and GRF components. Gait asymmetry was quantified using a standardized asymmetry index (AI) [47–50], defined as:

$$\text{AI}(\%)=|X_{\text{left}}-X_{\text{right}}|/(0.5\times (|X_{\text{left}}+X_{\text{right}}|)\times 100\%$$

Where X_left_ and X_right_ represent the values of the corresponding parameter on the left and right sides, respectively. A higher AI value indicates a greater discrepancy between the two limbs.

### 2.10. Statistical analysis

All statistical analyses were performed using Python 3.12 and R 4.3. Baseline characteristics and gait asymmetry outcomes were analyzed as follows. Continuous variables with normal distribution are presented as mean ± standard deviation (Mean ± SD), whereas non-normally distributed continuous variables are presented as median and interquartile range (IQR). Categorical variables are presented as frequencies and percentages.

Normality was assessed using the Shapiro–Wilk test. Homogeneity of variance was evaluated using Levene’s test. For continuous variables that met both normality and homogeneity of variance assumptions, between-group differences were examined using one-way analysis of variance (ANOVA), followed by Tukey’s post-hoc test. For normally distributed variables with unequal variances, Welch’s ANOVA followed by Games–Howell post-hoc comparisons was used. For non-normally distributed variables, the Kruskal–Wallis test was used, followed by pairwise Mann–Whitney U tests when appropriate. Categorical variables were compared using the chi-square test or Fisher’s exact test, as appropriate.

For gait asymmetry outcomes, most variables did not conform to a normal distribution; therefore, the Kruskal–Wallis test was used as the primary omnibus test to examine overall differences among the three frailty groups. Non-parametric tests were selected because they are less sensitive to distributional assumptions and are applicable to unequal group sizes. When a significant overall group effect was detected, post hoc pairwise comparisons were performed using Mann–Whitney U tests for the following contrasts: non-frail versus prefrail, non-frail versus frail, and prefrail versus frail. However, we acknowledge that non-parametric testing does not eliminate the reduced statistical power or potential estimate instability caused by the relatively small frail subgroup. To address multiple comparison issues, a hierarchical correction strategy was adopted. Each gait asymmetry feature was treated as a separate hypothesis family because the 51 gait asymmetry indices represent a structured and biomechanically correlated feature set derived from the same underlying construct of bilateral gait asymmetry, rather than a set of fully independent outcomes. Within each feature, the three post hoc pairwise comparisons were jointly adjusted using the Benjamini–Hochberg false discovery rate (FDR) procedure [51–53]. An FDR-adjusted p value < 0.05 was considered statistically significant. No additional global correction was applied across all 51 gait asymmetry outcomes. This decision was based on the exploratory aim of the study and the structured, biomechanically correlated nature of the gait asymmetry variables. The analysis was intended to characterize distributed patterns across predefined spatiotemporal, kinematic, and kinetic domains, rather than to test a single confirmatory hypothesis across all gait features. Therefore, treating all 51 outcomes as one independent hypothesis family would not fully reflect the structure of the data and could increase the risk of type II error. For non-parametric pairwise comparisons, Cliff’s delta (Δ) was used to quantify effect sizes based on all pairwise ordering combinations between two groups [54]. Effect size magnitude was interpreted using standard thresholds: negligible (|Δ| < 0.147), small (0.147–0.33), medium (0.33–0.474), and large (|Δ| ≥ 0.474). To further assess the stability of the effect-size estimates, 95% confidence intervals for Cliff’s delta were calculated using non-parametric bootstrap resampling with 5,000 iterations. These confidence intervals were used to evaluate the precision of the observed between-group differences, particularly for comparisons involving the smaller frail subgroup.

To evaluate the robustness of the primary findings to between-group differences in demographic and clinical characteristics, post hoc covariate-adjusted sensitivity analyses were conducted for the nine gait asymmetry outcomes that were significant in the primary analyses. For each outcome, a linear model was fitted to rank-transformed values, with frailty status as the primary explanatory variable and age, sex, body mass index, number of chronic conditions, and number of long-term medications included as covariates. Adjusted pairwise comparisons were performed among the non-frail, prefrail, and frail groups, with FDR correction applied to the three pairwise contrasts within each outcome.

### 2.11. Sample size consideration and sensitivity analysis

To improve methodological transparency, we conducted detectable effect-size analyses to evaluate the sensitivity of the current sample size. Because power estimation for non-parametric tests under multiple outcomes with FDR control depends on distributional assumptions and the unknown proportion of true effects, parametric approximations were used for interpretability. For the overall three-group comparison, a one-way ANOVA approximation showed that, with α = 0.05 and *N* = 150, the power to detect a medium effect size (Cohen’s *f* = 0.25) was approximately 0.78, and the minimum detectable effect size required to achieve 80% power was *f* ≈ 0.256, corresponding to η^2^ ≈ 0.062.

For pairwise comparisons, two-sample power approximations were used only to describe the sensitivity of the current sample size. The minimum detectable effects required for 80% power were *d* = 0.512 for non-frail versus prefrail, *d* = 0.741 for non-frail versus frail, and *d* = 0.694 for prefrail versus frail, corresponding approximately to Cliff’s delta values of 0.283, 0.400, and 0.376, respectively. These values were not used as the primary effect-size estimates, which were reported using Cliff’s delta with bootstrap 95% confidence intervals. These results indicate limited sensitivity for detecting small effects, particularly in comparisons involving the frail subgroup; therefore, non-significant frail-related findings should not be interpreted as evidence of no difference.

In addition, an exploratory alternative-grouping sensitivity analysis was performed by comparing frail participants with the combined non-frail/prefrail group. This analysis was used to evaluate whether the main frail-related differences were broadly consistent when the small frail subgroup was contrasted against all other participants.

## 3. Results

### 3.1. Participant flow and baseline characteristics

A total of 201 older adults were initially screened for eligibility. Of these, 38 were excluded because they did not meet the prespecified eligibility criteria, including a history of stroke (*n* = 10), previous joint replacement surgery (*n* = 8), marked lower-limb pain affecting natural walking (*n* = 14), or Parkinson’s disease (*n* = 6). An additional 13 individuals were excluded because of incomplete clinical data or refusal to continue gait testing. Ultimately, 150 participants were included in the final analysis, comprising 49 non-frail, 80 prefrail, and 21 frail participants. The baseline demographic and anthropometric characteristics of the three groups are summarized in Table 1. Overall, the groups were broadly comparable in most general characteristics, although several functional measures showed a gradual decline with increasing frailty severity. These baseline differences provide important context for the group-specific patterns of gait asymmetry observed in the subsequent analyses.

**Table 1.** Summary of baseline demographic and anthropometric characteristics.

Variable	Non-frail (*n* = 49)	Prefrail (*n* = 80)	Frail (*n* = 21)	P-value
Age (years)	68.04 ± 5.0	71.40 ± 5.82	74.57 ± 6.83	<0.001***
Sex				0.617
Female	29 (59.2%)	53 (66.2%)	12 (57.1%)	
Male	20 (40.8%)	27 (33.8%)	9 (42.9%)	
BMI (kg/m²)	23.67 ± 2.76	24.38 ± 3.31	24.21 ± 3.99	0.475
Education				0.889
Primary school	8	11	3	
Junior high school	10	20	5	
Senior high school	20	25	6	
College and above	11	24	7	
Comorbidities (count)	2.00 (1.00–3.00)	2.00 (1.00–3.00)	3.00 (1.00–4.00)	0.0886
Number of Medications	1.00 (0.00–2.00)	2.00 (0.00–3.00)	4.00 (2.00–5.00)	<0.001***
Nutrition status				0.092
Malnourished	0	3	3	
At risk	19	24	9	
Normal	30	52	9	
Psychological Status				0.459
Depressed	0	1	0	
Depressive tendency	5	9	5	
Normal	44	70	16	
Sleep Status				0.216
Insomnia	16	33	8	
Possible insomnia	7	16	7	
Normal	26	31	6	
Grip strength right (kg)	25.90 (21.70–32.90)	22.98 (18.20–27.92)	21.30 (15.80–24.30)	0.001**
TUGT(s)	9.57 (8.25–10.80)	10.32 (9.13–12.36)	12.91 (12.08–15.78)	<0.001***
SPPB				<0.001***
Poor	0	3	7	
Moderate	14	33	8	
Good	35	44	6	

Note: BMI: Body Mass Index; TUGT: Timed Up and Go Test; SPPB: Short Physical Performance Battery.

### 3.2. Overall gait asymmetry differences

Among the 51 gait asymmetry indices, 9 demonstrated significant between-group differences and showed significant FDR-adjusted pairwise contrasts (Table 2). These nine features were distributed across spatiotemporal, kinematic, and kinetic domains.

**Table 2.** Significant gait asymmetry features with raw p-values, FDR-adjusted p-values, and Cliff’s delta effect sizes.

Feature	Comparison	Raw_p	*P*	Cliffs_Delta
**Spatiotemporal parameter**				
Single Support AI	Non-frail vs Prefrail	0.011*	0.033*	−0.268 (−0.453,−0.067)
	Non-frail vs Frail	0.065†	0.097†	−0.281 (−0.561,0.022)
	Prefrail vs Frail	0.963	0.963	0.007 (−0.267,0.279)
Stride Length AI	Non-frail vs Prefrail	0.000**	0.001**	−0.388 (−0.567,−0.201)
	Non-frail vs Frail	0.014*	0.020*	−0.373 (−0.673,−0.057)
	Prefrail vs Frail	0.615	0.615	−0.072 (−0.382,0.232)
**Kinematic parameter**				
Peak Hip Flexion AI	Non-frail vs Prefrail	0.948	0.948	−0.007 (−0.209,0.201)
	Non-frail vs Frail	0.014*	0.025*	−0.374 (−0.631,−0.106)
	Prefrail vs Frail	0.017*	0.025*	−0.342 (−0.585,−0.082)
Peak Knee Flexion AI	Non-frail vs Prefrail	0.131	0.131	−0.159 (−0.370,0.052)
	Non-frail vs Frail	0.006**	0.019*	−0.415 (−0.691,−0.106)
	Prefrail vs Frail	0.038*	0.056†	−0.296 (−0.585,0.017)
Peak Hip External Rotation AI	Non-frail vs Prefrail	0.813	0.813	0.024 (−0.178,0.226)
	Non-frail vs Frail	0.017*	0.026*	−0.339 (−0.572,−0.083)
	Prefrail vs Frail	0.007**	0.022*	−0.366 (−0.585,−0.115)
Peak Foot External Rotation AI	Non-frail vs Prefrail	0.310	0.310	−0.107 (−0.305,0.098)
	Non-frail vs Frail	0.031*	0.092†	−0.328 (−0.596,−0.033)
	Prefrail vs Frail	0.182	0.273	−0.190 (−0.462,0.089)
Kinetic parameter				
Peak Knee Extension Moment AI	Non-frail vs Prefrail	0.079†	0.118	−0.183 (−0.383,0.014)
	Non-frail vs Frail	0.031*	0.094†	−0.325 (−0.603,−0.032)
	Prefrail vs Frail	0.296	0.296	−0.146 (−0.423,0.135)
Peak Knee Flexion Moment AI	Non-frail vs Prefrail	0.805	0.805	−0.026 (−0.229,0.179)
	Non-frail vs Frail	0.013*	0.027*	−0.375 (−0.65,−0.09)
	Prefrail vs Frail	0.018*	0.027*	−0.336 (−0.598,−0.058)
Peak Knee Eccentric Power AI	Non-frail vs Prefrail	0.898	0.898	−0.014 (−0.219,0.186)
	Non-frail vs Frail	0.024*	0.040*	−0.343 (−0.590,−0.078)
	Prefrail vs Frail	0.027*	0.040*	−0.315 (−0.536,−0.090)

Note: Pairwise comparisons of the nine significant gait asymmetry features using the Mann–Whitney U test. Raw p-values, *P* values (BH-FDR-adjusted *P* value) and Cliff’s delta (Δ) effect sizes. FDR correction was performed within each feature (three comparisons per feature). Effect size magnitude was interpreted as negligible (|Δ| < 0.147), small (0.147 ≤ |Δ| < 0.33), medium (0.33 ≤ |Δ| < 0.474), or large (|Δ| ≥ 0.474); Peak values derived from time-normalized waveforms (max or min depending on biomechanical meaning); AI = Asymmetry Index; *P < 0.05 and **P < 0.01; ^†^denotes trend-level differences (0.05 ≤ *P* < 0.10).

**Figure 1.**
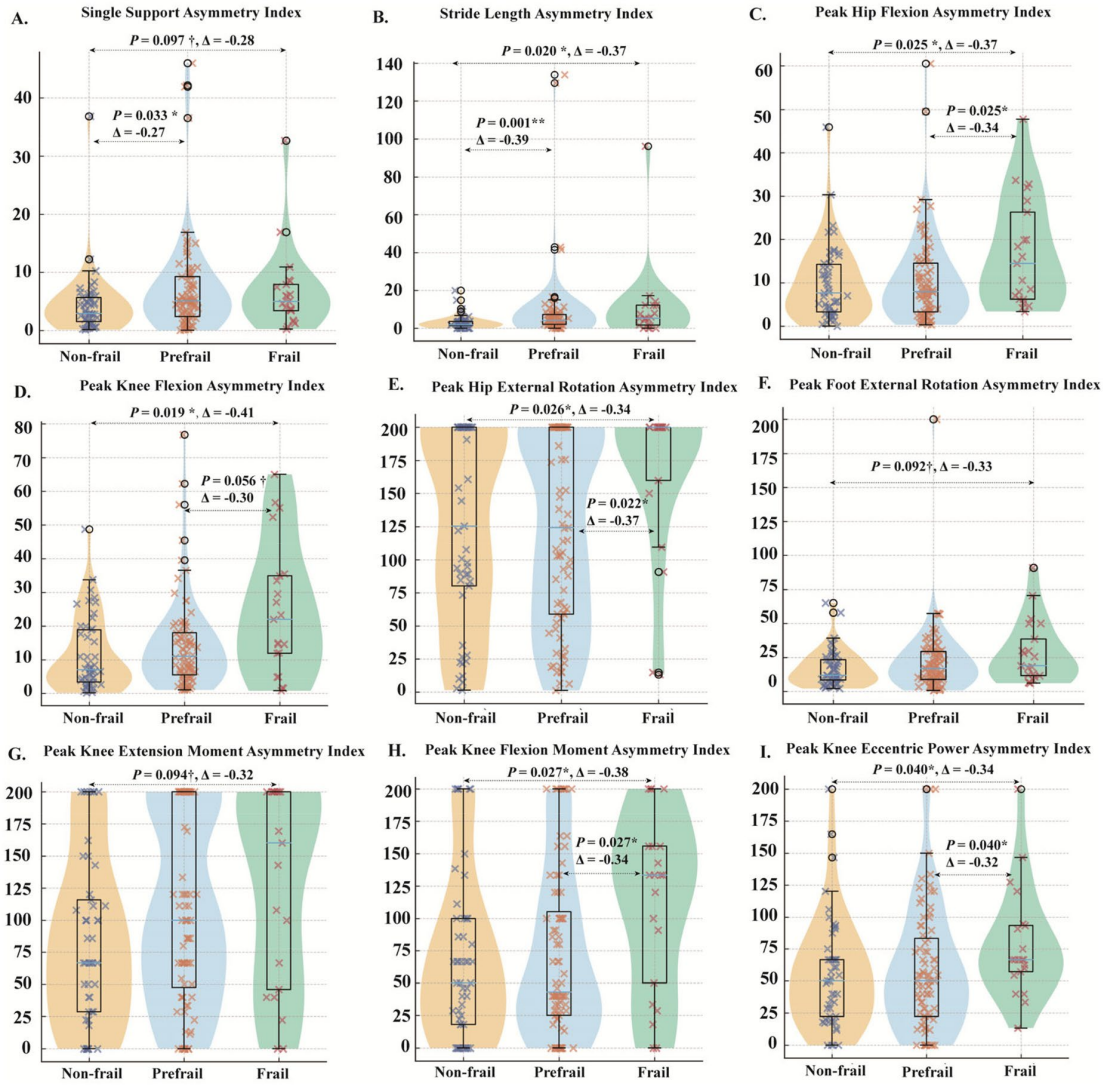
Raincloud plots of the nine gait asymmetry features that differed significantly across frailty-status groups (non-frail, prefrail, frail). Note: (A) Single support asymmetry index. (B) Stride length asymmetry index. (C) Peak hip flexion asymmetry index. (D) Peak knee flexion asymmetry index. (E) Peak hip external rotation asymmetry index. (F) Peak foot external rotation asymmetry index. (G) Peak knee extension moment asymmetry index. (H) Peak knee flexion moment asymmetry index. (I) Peak knee eccentric power asymmetry index. Higher asymmetry values indicate greater left–right gait imbalance. In each panel, the violin plot depicts the distribution, the boxplot summarizes the median and interquartile range, and dots represent individual participants. Pairwise comparisons were conducted using the Mann–Whitney U test, with p-values annotated as *P < 0.05 and *P < 0.01; ^†^denotes trend-level differences (0.05 ≤ *P* < 0.10). Cliff’s delta (Δ) is reported for each pairwise comparison to indicate the magnitude and direction of group differences.

**Figure 2.**
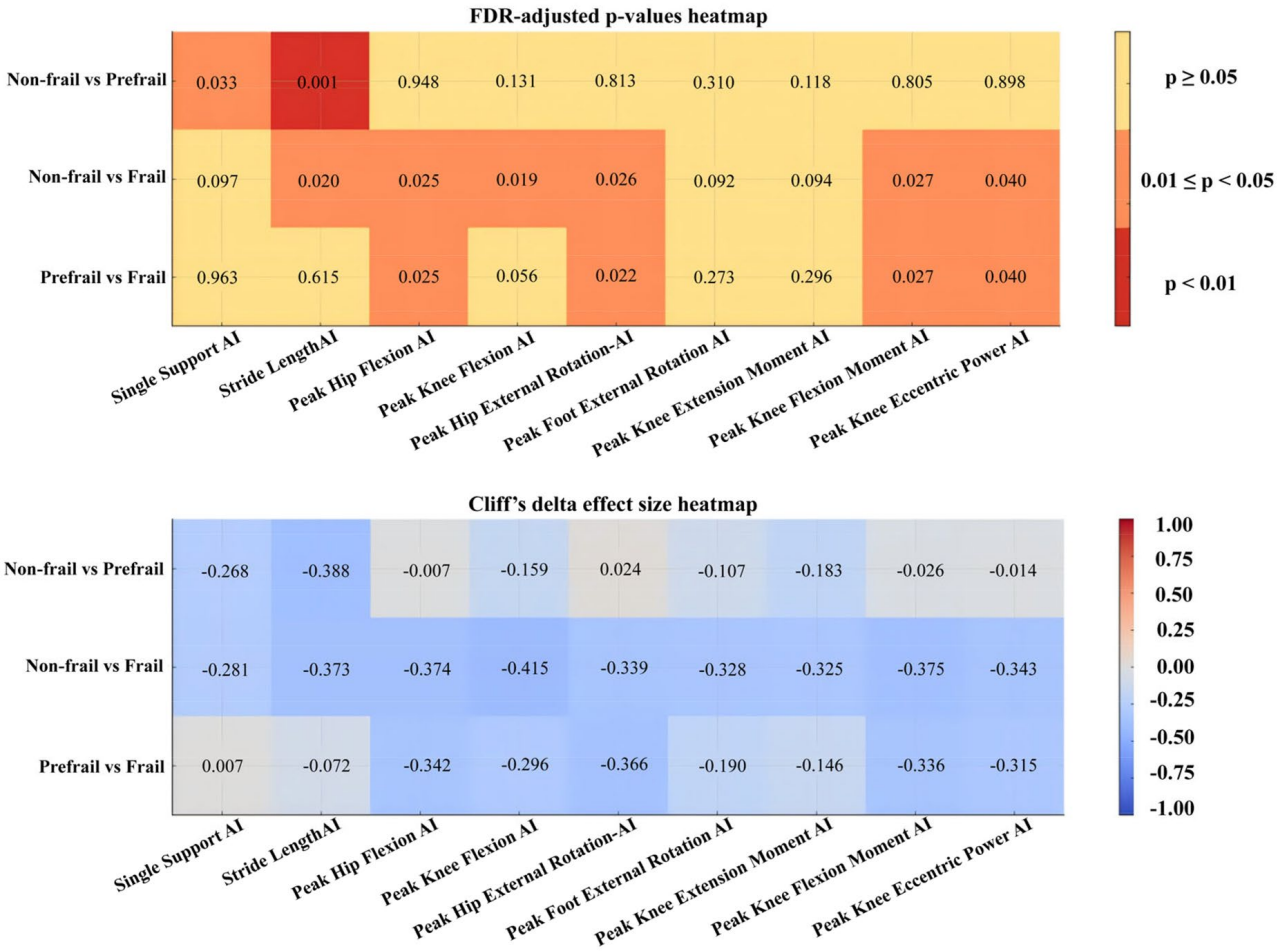
Vertical dual heatmap summarizing between-group differences in gait asymmetry indices. Note: The upper panel displays Benjamini–Hochberg FDR-adjusted p-values for the three pairwise comparisons (non-frail vs prefrail, non-frail vs frail, prefrail vs frail) using discrete threshold coloring. The lower panel shows the corresponding Cliff’s delta effect sizes on a continuous scale from −1 to +1, where positive values indicate greater asymmetry in the first group of each comparison and negative values indicate greater asymmetry in the second group; larger absolute values reflect larger between-group differences; AI: Asymmetry Index.

**Figure 3.**
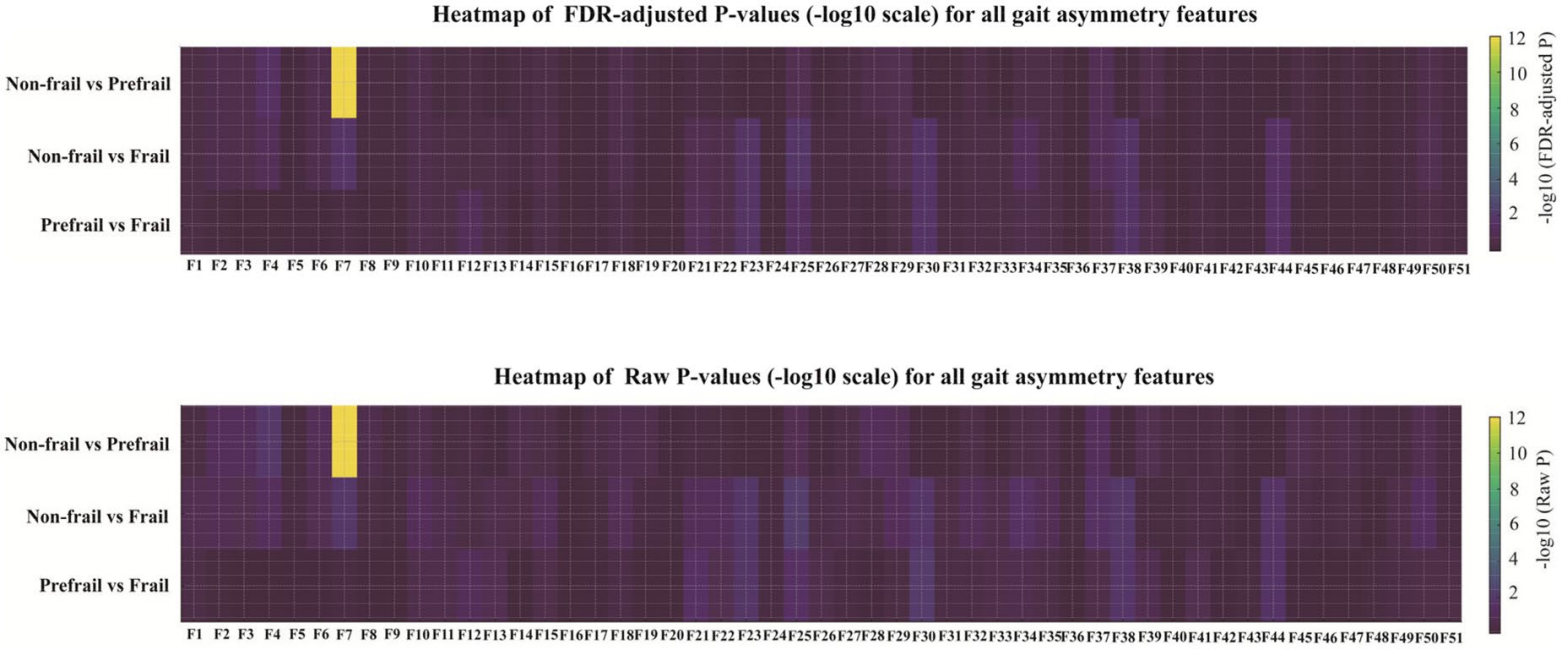
Heatmap of FDR-adjusted p-values (expressed as − log10) and Raw p-values (expressed as − log10) for all 51 gait asymmetry features. Note: Columns represent the 51 asymmetry parameters (full feature names are provided in Table S1), and rows represent the three pairwise group comparisons (Non-frail vs Prefrail, Non-frail vs Frail, and Prefrail vs Frail). Brighter colors indicate smaller p-values and stronger group differences.

Raincloud plots of the nine significant indices (Figure 1) show that asymmetry values generally tended to be higher in the prefrail and frail groups than in the non-frail group, with greater inter-individual variability particularly in the prefrail and frail groups. The vertical dual heatmap (Figure 2) provides a combined visualization of FDR-adjusted p-values and corresponding Cliff’s delta effect sizes for these features, allowing the statistical evidence and magnitude of between-group differences to be examined together. Figure 3 presents the raw and FDR-adjusted p-value patterns for all 51 gait asymmetry indices, providing an overview of the distribution of statistical evidence across the full set of analyzed gait features.

### 3.3. Non-frail vs. prefrail

In the comparison between the non-frail and prefrail groups, we observed between-group differences in selected gait asymmetry indices, with spatiotemporal parameters showing the clearest differences. The prefrail group showed markedly higher asymmetry in single-limb support (*p* = 0.033, Δ = −0.268, Figure 1A) and stride length (*p* = 0.001, Δ = −0.388, Figure 1B) than the non-frail group. In this comparison, significant pairwise differences were observed in single-support time and stride length asymmetry. No kinematic or kinetic asymmetry outcome showed a significant FDR-adjusted pairwise difference between the non-frail and prefrail groups. Thus, the non-frail versus prefrail comparison mainly showed feature-level differences within the spatiotemporal domain.

### 3.4. Non-frail vs. frail

In the non-frail versus frail comparison, significant pairwise differences were observed across multiple domains. In terms of spatiotemporal parameters, the frail group showed significantly greater stride length asymmetry (*p* = 0.020, Δ = −0.373, Figure 1B), and single-limb support asymmetry demonstrated a trend toward higher values (*p* = 0.097, Δ = −0.281, Figure 1A). At the kinematic level, asymmetry in hip flexion (*p* = 0.025, Δ = −0.374, Figure 1C), knee flexion (*p* = 0.019, Δ = −0.415, Figure 1D), and hip external rotation (*p* = 0.026, Δ = −0.339, Figure 1E) was significantly greater in the frail group than in the no-frail group, while foot external rotation asymmetry showed a trend toward higher values in the frail group (*p* = 0.092, Δ = −0.328, Figure 1F). In the kinetic domain, asymmetry in knee flexion moment (*p* = 0.027, Δ = −0.375, Figure 1H) and knee eccentric power (*p* = 0.040, Δ = −0.343, Figure 1I) was significantly elevated in the frail group, and knee extension moment asymmetry (*p* = 0.094, Δ = −0.325, Figure 1G) showed a tendency toward increase. Together, the non-frail versus frail comparison showed feature-level differences distributed across spatiotemporal, kinematic, and kinetic domains.

### 3.5. Prefrail vs. frail

In the comparison between the prefrail and frail groups, additional between-group differences were mainly observed at the joint level in terms of kinematic and kinetic asymmetry, whereas spatiotemporal parameters did not differ significantly between groups. There were no significant differences in single-limb support or stride length asymmetry (*p* > 0.05, Figure 1A, B), suggesting that prefrail and frail participants showed similar levels of spatiotemporal asymmetry, while frail participants showed greater differences in selected joint- and force-level asymmetry measures. Kinematically, the frail group showed significantly greater asymmetry in peak hip flexion (*p* = 0.025, Δ = −0.342, Figure 1C) and hip external rotation (*p* = 0.022, Δ = −0.366, Figure 1E) compared with the prefrail group, while knee flexion asymmetry demonstrated a trend toward higher values (*p* = 0.056, Δ = −0.296, Figure 1D). This pattern suggests that, compared with prefrail participants, frail participants showed greater asymmetry in selected hip/knee kinematic outcomes, including hip flexion and hip external rotation. On the kinetic side, asymmetry in knee flexion moment (*p* = 0.027, Δ = −0.336, Figure 1H) and knee eccentric power (*p* = 0.040, Δ = −0.315, Figure 1I) further increased in the frail group (Figure 1H, I). Taken together, the prefrail versus frail comparison mainly showed feature-level differences within kinematic and kinetic outcomes.

### 3.6. Covariate-adjusted sensitivity analysis

After adjustment for age, sex, body mass index, number of chronic conditions, and number of long-term medications (Table S2), prefrail participants retained greater single-support time asymmetry than non-frail participants (*p* = 0.046) and greater stride length asymmetry (*p* = 0.002). Frail participants also retained greater stride length asymmetry than non-frail participants (*p* = 0.035). In addition, knee flexion moment asymmetry remained greater in frail participants than in both non-frail and prefrail participants (All *p* = 0.041). The previously observed differences in other kinematic and kinetic asymmetry outcomes were attenuated after covariate adjustment. Overall, selected between-group differences in stride length, single-support time, and knee flexion moment asymmetry persisted after accounting for measured demographic and clinical covariates. However, the attenuation of several associations after adjustment suggests that age, sex, body composition, comorbidity burden, and long-term medication exposure may partly contribute to the observed between-group differences.

### 3.7. Additional robustness analyses related to group size imbalance

Additional analyses were performed to describe the effect-size precision of comparisons involving the relatively small frail subgroup (Table 2). Bootstrap 95% confidence intervals for Cliff’s delta were reported for the pairwise comparisons. For the non-frail versus frail comparison, the confidence intervals for stride length, peak hip flexion, peak knee flexion, peak hip external rotation, peak foot external rotation, peak knee extension moment, peak knee flexion moment, and peak knee eccentric power asymmetry remained in the same direction and did not include zero. The confidence interval for single-support time asymmetry included zero. For the prefrail versus frail comparison, the confidence intervals for peak hip flexion, peak hip external rotation, peak knee flexion moment, and peak knee eccentric power asymmetry remained in the same direction and did not include zero. The confidence intervals for single-support time, stride length, peak knee flexion, peak foot external rotation, and peak knee extension moment asymmetry included zero. In general, confidence intervals for comparisons involving the frail group were relatively wide, reflecting the smaller sample size of the frail subgroup.

In the exploratory alternative-grouping sensitivity analysis, frail participants were compared with the combined non-frail/prefrail group (Table S3). After FDR correction, frail participants showed greater asymmetry in peak hip flexion, peak knee flexion, peak hip external rotation, peak knee flexion moment, and peak knee eccentric power. Given the small frail subgroup, these results were reported as additional exploratory analyses.

### 3.8. Correlation-structure analysis of gait asymmetry outcomes

To empirically describe the correlation structure and potential redundancy among gait asymmetry outcomes, pairwise Spearman correlation coefficients were calculated across the 51 gait asymmetry indices. Hierarchical clustering was then performed using average linkage based on correlation distance, defined as 1 − |ρ|. This analysis was descriptive and was used to examine whether gait asymmetry variables showed identifiable clustering patterns across biomechanical domains.

Across the 1275 pairwise correlations among the 51 gait asymmetry outcomes, the median absolute Spearman correlation coefficient was 0.071 (IQR, 0.033–0.129), and the mean absolute correlation coefficient was 0.099. Sixty-two variable pairs (4.9%) showed |ρ| ≥ 0.30, 13 pairs (1.0%) showed |ρ| ≥ 0.50, and one pair (0.1%) showed |ρ| ≥ 0.70. The maximum absolute correlation coefficient was 0.873 (Table S4). The correlation matrix structure are shown in Figure S1.

Hierarchical clustering identified several local clusters with biomechanical interpretability, including clusters involving spatiotemporal parameters, sagittal-plane hip/knee kinematic measures, foot progression/external rotation measures, global gait deviation indices, and selected ankle-related measures (Table S5). These findings indicate that the gait asymmetry outcomes were not fully independent, although extensive high correlation across all variables was not observed. Therefore, individual significant features were interpreted as exploratory feature-level associations within a structured gait asymmetry dataset, rather than as isolated independent effects.

## 4. Discussion

This cross-sectional study used three-dimensional gait analysis to describe feature-level gait asymmetry patterns across predefined spatiotemporal, kinematic, and kinetic domains in non-frail, prefrail, and frail older adults. Gait asymmetry patterns differed across frailty-status groups. Compared with non-frail participants, prefrail participants showed differences primarily in selected spatiotemporal asymmetry measures, whereas frail participants exhibited differences involving selected spatiotemporal, hip–knee kinematic, and knee kinetic asymmetry measures. These findings suggest that multidimensional gait asymmetry assessment may provide complementary biomechanical information for characterizing frailty-associated gait impairment.

### 4.1. Frailty-status-specific patterns of gait asymmetry

In the prefrail group, selected gait asymmetry differences were observed, particularly in spatiotemporal parameters such as single limb support time and stride length. These measures reflect the organization of gait rhythm and the distribution of loading between the two lower limbs. These between-group differences suggest that prefrail status was mainly associated with spatiotemporal asymmetry, whereas marked joint-level kinematic or kinetic asymmetry was less evident in this group. This is consistent with clinical observations that prefrail older adults may present with reduced walking stability, loss of confidence, and a subjective sense of poorer coordination [55]. In contrast, the frail group exhibited a broader range of asymmetry involving multiple domains. Beyond spatiotemporal characteristics, asymmetry was also observed in kinematic measures such as sagittal-plane hip and knee flexion angles and transverse-plane rotations, as well as in kinetic measures including knee flexion and extension moments and eccentric power. This cross-sectional pattern indicates that the distribution of feature-level gait asymmetry differences varied across frailty-status groups, with prefrail participants showing differences mainly in spatiotemporal asymmetry and frail participants showing additional differences in kinematic and kinetic asymmetry outcomes. However, this broader pattern observed in the frail group should be interpreted cautiously because the frail subgroup was relatively small and some effect-size estimates showed wide confidence intervals. In addition, because this study is cross-sectional, the between-group differences reflect comparisons among individuals at different frailty strata and should not be interpreted as within-individual progression over time. Nevertheless, the observed distribution of gait asymmetry differences across predefined biomechanical domains may provide complementary descriptive information for characterizing frailty-related functional status and offers a rationale for future longitudinal validation.

### 4.2. Combined effects of muscle weakness, reduced neuromuscular control, and compensatory strategies

The gait asymmetry pattern observed in this study is highly consistent with current understanding of neuromuscular alterations in frailty. On the one hand, frailty related declines in muscle mass and strength [56–60] reduce the ability of the lower limbs to generate symmetrical force during push-off and shock absorption, particularly at high demand joints such as the hip and knee that are responsible for support and propulsion. This leads to an imbalance in how the two limbs share load and generate force [61], which is expressed at the kinetic level as increased asymmetry in joint moments and joint power [62,63]. In our study, asymmetry in knee extensor moment, knee flexor moment and eccentric knee power was particularly pronounced, further supporting this mechanism. Sarcopenia and delayed neuromuscular activation in frail individuals may both contribute to side to side differences in how mechanical load is managed [58,64]. We also found that kinetic asymmetry indices exhibited more pronounced between-group differences and larger effect sizes, suggesting that they may provide additional information for characterizing frailty-status differences. This is consistent with previous findings that kinetic parameters directly reflect muscle force production, energy absorption and neuromuscular control, and may provide information complementary to kinematic or spatiotemporal measures in the assessment of frailty-related functional impairment [65]. On the other hand, frailty and age-related declines in motor control, such as reduced precision of muscle activation timing, impaired regulation of force output and diminished adaptability of gait patterns, can weaken an individual’s ability to maintain a symmetric gait rhythm and trajectory [59,63,66]. The increased asymmetry in hip and knee flexion and in lower limb rotational angles observed in frail older adults is likely to reflect a less coordinated movement strategy [67], in which one limb assumes a greater share of stabilization or propulsion, while the contralateral limb operates in a more passive or compensatory manner [68]. On this basis, compensatory behaviours may further amplify asymmetry. Frail older adults often unconsciously shift their weight toward the stronger or more stable limb, shorten stance time on the contralateral side, or adopt more asymmetric step lengths and foot placement patterns in order to maintain balance [49]. These compensatory patterns may help maintain balance during walking; however, whether they contribute to subsequent side-to-side differences in loading and motor control requires longitudinal confirmation. Taken together, differences in muscle strength, neuromuscular control, and compensatory movement strategies may partly explain the higher gait asymmetry observed in frail participants relative to the other groups, although causal relationships cannot be determined from the present cross-sectional data.

### 4.3. Implications for future assessment and rehabilitation research

The findings of this study may have implications for the future assessment and rehabilitation of frailty-associated gait impairment. Differences in spatiotemporal asymmetry, particularly stride length and single-support time asymmetry, were observed in prefrail participants compared with non-frail participants. These measures may therefore represent candidate features for further evaluation in more accessible community-based assessment approaches. Compared with traditional questionnaires or scales, gait metrics can be obtained objectively and may provide complementary information on walking performance. Although laboratory-based three-dimensional gait analysis is not routinely available in many clinical or community settings because of equipment cost, technical complexity, and the need for specialized personnel, it may serve as a reference method for identifying biomechanically informative asymmetry patterns. Future studies should determine whether a smaller set of informative and non-redundant measures can be reliably approximated using wearable inertial sensors, pressure-sensitive insoles, or portable sensor technologies.

Compared with non-frail and prefrail participants, frail participants showed additional differences in selected hip–knee kinematic and knee kinetic asymmetry measures. These findings may provide hypotheses for future rehabilitation research regarding the potential relevance of multi-joint coordination, limb symmetry, knee support, and propulsion-related function in frailty-status characterization. For example, asymmetry in hip and knee flexion and rotational kinematics may warrant further investigation in relation to coordination- and symmetry-oriented training, whereas asymmetry in knee flexion moment and eccentric power may warrant evaluation in relation to interventions targeting knee support, power generation, motor control, postural control, and gait retraining. However, the present cross-sectional findings do not establish that these gait characteristics are modifiable treatment targets or that interventions directed at these features will improve clinical outcomes.

Gait asymmetry measures may also be considered as potential objective outcomes in future intervention studies. Whether they are more sensitive than conventional clinical scales or global gait measures for detecting meaningful rehabilitation-related change remains to be determined. Prior evidence suggests that gait asymmetry is associated with impaired stability and elevated fall risk [69–71]. However, this study did not collect fall history or prospective fall outcomes and therefore cannot draw direct conclusions regarding fall risk. The observed differences in spatiotemporal, kinematic, and kinetic asymmetry patterns should not be interpreted as evidence of risk stratification ability, prognostic value, or frailty progression.

Overall, multidimensional gait asymmetry assessment provides complementary biomechanical information for characterizing frailty-associated motor impairment. Before translation into routine frailty screening, rehabilitation monitoring, or individualized intervention planning can be supported, further longitudinal studies, external validation, feasibility testing, and evaluation of clinically meaningful thresholds are required.

### 4.4. Strengths and limitations

This study has several strengths. First, we employed a comprehensive set of gait asymmetry indices spanning spatiotemporal parameters as well as multi-joint lower-limb kinematics and kinetics, enabling a multidimensional characterization of frailty-related gait alterations. Compared with traditional approaches that focus primarily on spatiotemporal measures, our analysis further incorporated joint angles, joint moments, and joint power, which enabled a broader descriptive assessment of inter-joint coordination and mechanical output across frailty-status groups. Second, we applied rigorous statistical procedures, including false discovery rate (FDR) correction and Cliff’s delta effect size estimation, which enhances the robustness of the findings and allows clearer distinction between statistical significance and the magnitude of group differences. Third, by including non-frail, prefrail, and frail older adults, we captured the frailty spectrum and were able to describe gait asymmetry patterns across frailty-status groups.

However, several limitations should be acknowledged. First, the cross-sectional design precludes causal inference; the between-group gradients across frailty strata should not be interpreted as within-individual progression. Longitudinal studies are needed to clarify temporal ordering and to test whether spatiotemporal asymmetry and multi-joint kinematic or kinetic asymmetry change over time within individuals. Second, this was a single-center study involving participants from one hospital and its surrounding community within a single geographical region. Differences in demographic, cultural, healthcare, and environmental factors may influence gait and frailty-related movement patterns. Therefore, the generalizability of these findings to other regions or healthcare settings may be limited and requires confirmation in multicenter, geographically diverse populations.Third, residual confounding cannot be completely ruled out. Although participants with diagnosed dementia, Parkinson’s disease, stroke with residual deficits, other neurological disorders, major orthopedic conditions, and pain conditions clearly affecting gait or gait symmetry were excluded according to prespecified eligibility criteria, subclinical neurological or neurocognitive abnormalities may still have been present. In particular, preclinical Alzheimer’s disease, vascular cognitive impairment, or other subtle cognitive changes may affect motor control and gait before the clinical onset of dementia. Similarly, mild-to-moderate musculoskeletal conditions, chronic pain, and imperfect clinical screening may have introduced residual confounding or misclassification. After adjustment for age, sex, body mass index, number of chronic conditions, and long-term medication use, selected differences in stride length, single-support time, and knee flexion moment asymmetry remained, suggesting that these associations were not fully explained by the measured demographic and clinical factors. However, several other kinematic and kinetic differences were attenuated after adjustment, indicating that these factors may have contributed to the observed gait asymmetry patterns. Accordingly, the findings should be interpreted as frailty-status-associated gait asymmetry patterns in a real-world older population, rather than evidence of an independent causal effect of frailty. Future longitudinal studies incorporating standardized cognitive assessments and more detailed neurological screening are needed to better distinguish the independent and interactive contributions of frailty, neurocognitive status, and other age-related conditions to gait asymmetry. Fourth, laboratory-based three-dimensional motion capture may not fully reflect habitual gait in daily life; combining high-precision gait analysis with real-world wearable monitoring may improve ecological validity and clinical feasibility. Fifth, the frail group was relatively small (*n* = 21) compared with the non-frail and prefrail groups. Additional detectable effect-size analyses showed that comparisons involving the frail subgroup required relatively large effect sizes to achieve adequate statistical power, indicating limited sensitivity for small frail-related differences. Bootstrap confidence intervals for Cliff’s delta also suggested that some frail-related effect-size estimates were imprecise. Although non-parametric tests were used because most gait asymmetry variables were not normally distributed and because these methods are applicable to unequal group sizes, they do not resolve limitations related to reduced power or estimate instability. Therefore, non-significant findings involving the frail group should not be interpreted as evidence of no difference, and statistically significant findings involving this group should be regarded as exploratory. Larger studies with more balanced frailty-status groups are needed to confirm the robustness of these findings. Finally, the high-dimensional nature, correlation structure, and multiple testing burden of the gait asymmetry dataset should be considered when interpreting the findings. Although the inclusion of spatiotemporal, kinematic, and kinetic variables provided a comprehensive description of gait asymmetry, the 51 gait asymmetry outcomes were not fully independent. The additional correlation-structure analysis showed that most pairwise correlations were low, indicating that the dataset was not composed of a small number of uniformly redundant variables. However, several biomechanically interpretable local clusters were identified, suggesting that some outcomes may contain overlapping information within related gait domains. Therefore, individual significant features should not be interpreted as isolated independent effects, but rather as exploratory feature-level associations within a structured gait asymmetry dataset. In addition, the multiple testing issue arising from the full set of gait asymmetry outcomes should also be considered. Although we adopted a hierarchical correction strategy in which the three planned pairwise comparisons within each gait asymmetry feature were adjusted using the BH FDR procedure, no additional global correction was applied across all 51 outcomes. This decision was based on the exploratory aim of the study and the structured, biomechanically correlated nature of the gait asymmetry variables. The analysis was intended to characterize distributed biomechanical patterns across predefined spatiotemporal, kinematic, and kinetic domains, rather than to conduct a confirmatory test of a single global outcome family. Therefore, treating all 51 outcomes as fully independent hypotheses would not fully reflect the structure of the data and could increase the risk of type II error. However, this analytical decision also limits the strength of inference. Because the analysis did not control the global error rate across all 51 outcomes, the possibility of false-positive findings cannot be excluded. Accordingly, the reported significant findings should be interpreted as exploratory, feature-level associations rather than independently confirmed or clinically actionable markers. The present study did not aim to derive an optimal or non-redundant set of predictive markers through dimensionality reduction or external validation. Future studies with larger independent cohorts should incorporate pre-specified primary asymmetry outcomes, dimensionality reduction, multivariable modeling, and external validation to identify the most informative, stable, and non-redundant gait asymmetry markers associated with frailty status.

## 5. Conclusion

This study described cross-sectional, feature-level gait asymmetry patterns across non-frail, prefrail, and frail older adults using three-dimensional gait analysis. The observed between-group differences were distributed across predefined spatiotemporal, kinematic, and kinetic domains. In prefrail older adults, differences were mainly observed in spatiotemporal asymmetry, including stride length and single-support time. In frail older adults, feature-level differences were more broadly distributed and involved hip/knee kinematic and knee kinetic asymmetry outcomes, including hip and knee flexion, hip external rotation, knee flexion moment, and knee eccentric power.

These findings suggest that gait asymmetry may provide complementary biomechanical information for characterizing frailty-related gait impairment. However, given the cross-sectional design, the high-dimensional and correlated nature of the gait asymmetry dataset, and the relatively small frail subgroup, the findings should be interpreted as exploratory domain-informed patterns rather than evidence of temporal progression, independent predictive markers, or clinically actionable targets. Future studies with longitudinal follow-up, larger and more balanced cohorts, dimensionality reduction, external validation, and real-world wearable monitoring are needed to clarify the prognostic utility and clinical applicability of gait asymmetry measures in frailty assessment and rehabilitation.

## Supplementary Material

Supplementary File 1.docx

## Data Availability

The datasets generated and/or analyzed during the current study are not publicly available due to participant privacy and ethical restrictions. De-identified data may be made available from the corresponding author upon reasonable request, subject to institutional approval, data use agreement, and compliance with relevant ethical requirements.
