# Supplementary material for: Gait asymmetry across frailty-status groups in older adults: clinical and biomechanical evidence from 3D gait analysis
Source: Ann Med. 2026 Jul 14;58(1):2702656. doi: 10.1080/07853890.2026.2702656 (PMC13371482; doi:10.1080/07853890.2026.2702656)
Supplement: Supplementary File 1.docx [file IANN_A_2702656_SM0869.docx]

**Supplementary File 1**

**Table S1. Full names and abbreviations of the 51 gait asymmetry features.**

| **Feature ID** | **Full Name** | **Abbreviation** |
| --- | --- | --- |
| F1 | Stride Time Asymmetry Index (%) | STAI |
| F2 | Stance Phase Asymmetry Index (%) | SPAI |
| F3 | Swing Phase Asymmetry Index (%) | SWAI |
| F4 | Single Support Asymmetry Index (%) | SSAI |
| F5 | Double Support Asymmetry Index (%) | DSAI |
| F6 | Step Length Asymmetry Index (%) | SLAI |
| F7 | Stride Length Asymmetry Index (%) | StLAI |
| F8 | Gait Profile Score Asymmetry Index (%) | GPS-AI |
| F9 | Pelvic Obliquity Asymmetry Index (%) | PO-AI |
| F10 | Pelvic Tilt Asymmetry Index (%) | PT-AI |
| F11 | Pelvic Rotation Asymmetry Index (%) | PR-AI |
| F12 | Hip Adduction–Abduction Asymmetry Index (%) | HAA-AI |
| F13 | Hip Flexion–Extension Asymmetry Index (%) | HFE-AI |
| F14 | Hip Rotation Asymmetry Index (%) | HR-AI |
| F15 | Knee Flexion–Extension Asymmetry Index (%) | KFE-AI |
| F16 | Ankle Dorsiflexion–Plantarflexion Asymmetry Index (%) | ADP-AI |
| F17 | Foot Progression Angle Asymmetry Index (%) | FPA-AI |
| F18 | Gait Deviation Index Asymmetry Index (%) | GDI-AI |
| F19 | Peak Hip Adduction Asymmetry Index (%) | Peak HipAdd-AI |
| F20 | Peak Hip Abduction Asymmetry Index (%) | Peak HipAbd-AI |
| F21 | Peak Knee Varus Asymmetry Index (%) | Peak KneeVar-AI |
| F22 | Peak Knee Valgus Asymmetry Index (%) | Peak KneeVal-AI |
| F23 | Peak Hip Flexion Asymmetry Index (%) | Peak HipFlex-AI |
| F24 | Peak Hip Extension Asymmetry Index (%) | Peak HipExt-AI |
| F25 | Peak Knee Flexion Asymmetry Index (%) | Peak KneeFlex-AI |
| F26 | Peak Knee Extension Asymmetry Index (%) | Peak KneeExt-AI |
| F27 | Peak Ankle Dorsiflexion Asymmetry Index (%) | Peak AnkleDF-AI |
| F28 | Peak Ankle Plantarflexion Asymmetry Index (%) | Peak AnklePF-AI |
| F29 | Peak Hip Internal Rotation Asymmetry Index (%) | Peak HipIR-AI |
| F30 | Peak Hip External Rotation Asymmetry Index (%) | Peak HipER-AI |
| F31 | Peak Knee Internal Rotation Asymmetry Index (%) | Peak KneeIR-AI |
| F32 | Peak Knee External Rotation Asymmetry Index (%) | Peak KneeER-AI |
| F33 | Peak Foot Internal Rotation Asymmetry Index (%) | Peak FootIR-AI |
| F34 | Peak Foot External Rotation Asymmetry Index (%) | Peak FootER-AI |
| F35 | Peak Hip Extension Moment Asymmetry Index (%) | Peak HipExtMom-AI |
| F36 | Peak Hip Flexion Moment Asymmetry Index (%) | Peak HipFlexMom-AI |
| F37 | Peak Knee Extension Moment Asymmetry Index (%) | Peak KneeExtMom-AI |
| F38 | Peak Knee Flexion Moment Asymmetry Index (%) | Peak KneeFlexMom-AI |
| F39 | Peak Ankle Plantarflexion Moment Asymmetry Index (%) | Peak AnklePFMom-AI |
| F40 | Peak Ankle Dorsiflexion Moment Asymmetry Index (%) | Peak AnkleDFMom-AI |
| F41 | Peak Hip Concentric Power Asymmetry Index (%) | Peak HipConPwr-AI |
| F42 | Peak Hip Eccentric Power Asymmetry Index (%) | Peak HipEccPwr-AI |
| F43 | Peak Knee Concentric Power Asymmetry Index (%) | Peak KneeConPwr-AI |
| F44 | Peak Knee Eccentric Power Asymmetry Index (%) | Peak KneeEccPwr-AI |
| F45 | Peak Ankle Concentric Power Asymmetry Index (%) | Peak AnkleConPwr-AI |
| F46 | Peak Ankle Eccentric Power Asymmetry Index (%) | Peak AnkleEccPwr-AI |
| F47 | Peak Vertical Ground Reaction Force Asymmetry Index (%) | Peak vGRF-AI |
| F48 | Peak Anterior Ground Reaction Force Asymmetry Index (%) | Peak aGRF-AI |
| F49 | Peak Posterior Ground Reaction Force Asymmetry Index (%) | Peak pGRF-AI |
| F50 | Peak Medial Ground Reaction Force Asymmetry Index (%) | Peak mGRF-AI |
| F51 | Peak Lateral Ground Reaction Force Asymmetry Index (%) | Peak lGRF-AI |

**Table S2. Comparison of gait asymmetry indices across frailty-status groups after adjustment for demographic and clinical covariates.**

| **Feature** | **Comparison** | **Raw_p** | ***P*** |
| --- | --- | --- | --- |
| **Spatiotemporal parameter** | | | |
| Single Support AI | Non-frail vs Prefrail | 0.015***** | 0.046***** |
|  | Non-frail vs Frail | 0.076**†** | 0.114 |
|  | Prefrail vs Frail | 0.818 | 0.818 |
| Stride Length AI | Non-frail vs Prefrail | 0.001****** | 0.002****** |
|  | Non-frail vs Frail | 0.023***** | 0.035***** |
|  | Prefrail vs Frail | 0.964 | 0.964 |
| **Kinematic parameter** | | | |
| Peak Hip Flexion AI | Non-frail vs Prefrail | 0.015***** | 0.046***** |
|  | Non-frail vs Frail | 0.076**†** | 0.114 |
|  | Prefrail vs Frail | 0.818 | 0.818 |
| Peak Knee Flexion AI | Non-frail vs Prefrail | 0.001****** | 0.002****** |
|  | Non-frail vs Frail | 0.023***** | 0.035***** |
|  | Prefrail vs Frail | 0.964 | 0.964 |
| Peak Hip External Rotation AI | Non-frail vs Prefrail | 0.015***** | 0.046***** |
|  | Non-frail vs Frail | 0.076**†** | 0.114 |
|  | Prefrail vs Frail | 0.818 | 0.818 |
| Peak Foot External Rotation AI | Non-frail vs Prefrail | 0.001****** | 0.002****** |
|  | Non-frail vs Frail | 0.023***** | 0.035***** |
|  | Prefrail vs Frail | 0.964 | 0.964 |
| **Kinetic parameter** | | | |
| Peak Knee Extension Moment AI | Non-frail vs Prefrail | 0.151 | 0.226 |
|  | Non-frail vs Frail | 0.144 | 0.226 |
|  | Prefrail vs Frail | 0.543 | 0.543 |
| Peak Knee Flexion Moment AI | Non-frail vs Prefrail | 0.803 | 0.803 |
|  | Non-frail vs Frail | 0.028***** | 0.041***** |
|  | Prefrail vs Frail | 0.020***** | 0.041***** |
| Peak Knee Eccentric Power AI | Non-frail vs Prefrail | 0.847 | 0.847 |
|  | Non-frail vs Frail | 0.076**†** | 0.113 |
|  | Prefrail vs Frail | 0.031***** | 0.092**†** |

**Note:** Gait asymmetry indices were evaluated using rank-transformed ANCOVA-type linear models, with adjustment for age, sex, body mass index, number of chronic conditions, and long-term medication use. Adjusted pairwise comparisons were performed among the non-frail, prefrail, and frail groups. *P* values represent Benjamini–Hochberg false discovery rate (BH-FDR)-adjusted P values for the three pairwise comparisons within each gait asymmetry outcome.***** *P* < 0.05 and ****** *P* < 0.01; **†** denotes trend-level differences (0.05 ≤ *P* < 0.10).

**Table S3. Exploratory sensitivity analysis comparing frail participants with the combined non-frail & prefrail group.**

| **Outcome** | ***P*** | **Cliff’s δ (95% CI)** |
| --- | --- | --- |
| Single Support AI | 0.455 | 0.102 (-0.166, 0.360) |
| Stride Length AI | 0.193 | 0.186 (-0.112, 0.476) |
| Peak Hip Flexion AI | 0.028***** | 0.354 (0.108, 0.588) |
| Peak Knee Flexion AI | 0.028***** | 0.341 (0.056, 0.615) |
| Peak Hip External Rotation AI | 0.028***** | 0.356 (0.116, 0.567) |
| Peak Foot External Rotation AI | 0.113 | 0.243 (-0.018, 0.489) |
| Peak Knee Extension Moment AI | 0.145 | 0.214 (-0.047, 0.476) |
| Peak Knee Flexion Moment AI | 0.028***** | 0.351 (0.075, 0.599) |
| Peak Knee Eccentric Power AI | 0.030***** | 0.326 (0.109, 0.521) |

**Note:** *P* values represent Benjamini–Hochberg false discovery rate (BH-FDR)-adjusted P values for the three pairwise comparisons within each gait asymmetry outcome.***** *P* < 0.05.

**Table S4. Summary of correlation-structure and clustering analysis of gait asymmetry outcomes.**

| **Component** | **Result** |
| --- | --- |
| Number of gait asymmetry outcomes | 51 |
| Number of pairwise correlations | 1275 |
| Correlation method | Spearman correlation |
| Clustering method | Average-linkage hierarchical clustering |
| Correlation distance | 1 − \|ρ\| |
| Median absolute Spearman ρ | 0.071 |
| IQR of absolute Spearman ρ | 0.033–0.129 |
| Mean absolute Spearman ρ | 0.099 |
| Maximum absolute Spearman ρ | 0.873 |
| Variable pairs with \|ρ\| ≥ 0.30 | 62 / 1275, 4.9% |
| Variable pairs with \|ρ\| ≥ 0.50 | 13 / 1275, 1.0% |
| Variable pairs with \|ρ\| ≥ 0.70 | 1 / 1275, 0.1% |

**Table S5. Biomechanically interpretable local clusters identified by hierarchical clustering.**

| **Cluster** | **Variables** | **Biomechanical interpretation** |
| --- | --- | --- |
| Cluster 1 | SPAI,  SWAI,  SSAI | Spatiotemporal asymmetry-related measures |
| Cluster 2 | KneeVarMAX-AI, KneeValMAX-AI,  HipIRMAX-AI | Coronal-plane and rotational control-related measures |
| Cluster 3 | HipFlexMAX-AI, KneeFlexMAX-AI, KneeExtMAX-AI | Sagittal-plane hip/knee kinematic measures |
| Cluster 4 | ADP-AI,  AnkleDFMAX-AI | Ankle dorsiflexion-related measures |
| Cluster 5 | FPA-AI,  FootERMAX-AI | Foot progression/external rotation-related measures |
| Cluster 6 | GPS-AI,  GDI-AI | Global gait deviation-related indices |
| Cluster 7 | HFE-AI,  HipExtMAX-AI | Hip flexion-extension-related measures |

**Figure S1. Spearman correlation heatmap of the 51 gait asymmetry outcomes.**

Note: Variables were ordered according to average-linkage hierarchical clustering based on correlation distance, defined as 1 − |ρ|. Red indicates positive correlations, blue indicates negative correlations, and stronger color intensity indicates larger correlation magnitude.
